# Sarcoptic mange in Felidae: does *Sarcoptes scabiei* var.* felis *exist? A first molecular study[Fn FN1]

**DOI:** 10.1051/parasite/2023012

**Published:** 2023-03-31

**Authors:** Barbara Moroni, Francesco Albanese, Anna Rita Molinar Min, Mario Pasquetti, Jacques Guillot, Simone Roberto Rolando Pisano, Marie-Pierre Ryser-Degiorgis, Silvia Rüfenacht, Dominique Gauthier, David Cano-Terriza, Dino Scaravelli, Luca Rossi, Andrea Peano

**Affiliations:** 1 Department of Veterinary Sciences, University of Turin Largo Braccini 2 10095 Grugliasco Italy; 2 Istituto Zooprofilattico Sperimentale del Piemonte Liguria e Val d’Aosta, Via Bologna 148 10154 Torino Italy; 3 Centro Dermatologico Veterinario Toscano Via Romana, 4 52100 Arezzo Italy; 4 Mylav Private Veterinary Laboratory Via Sirtori, 9 20017 Passirana di Rho-Milano Italy; 5 Department of Dermatology-Parasitology-Mycology Oniris 44300 Nantes France; 6 Institute for Fish and Wildlife Health (FIWI), Department of Infectious Diseases and Pathobiology, Vetsuisse Faculty, University of Bern Laenggassstrasse 122, PO Box 3001 Bern Switzerland; 7 Dermavet, Tierklinik Aarau-West 5036 Oberentfelden Switzerland; 8 Laboratoire Départemental Vétérinaire des Hautes-Alpes (LDVHA 05) 05000 Gap France; 9 Department of Animal Health, UIC ENZOEM, Animal Health and Zoonosis Research Group (GISAZ), University of Cordoba 14014 Córdoba Spain; 10 CIBERINFEC, ISCIII-CIBER de Enfermedades Infecciosas, Instituto de Salud Carlos III 28029 Madrid Spain; 11 Department of Biological, Geological, and Environmental Sciences, University of Bologna via Selmi 3 40126 Bologna Italy

**Keywords:** Sarcoptic mange, Scabies, Felid, Carnivore, Host-specificity, Genetic structure

## Abstract

Domestic and wild felids are considered suitable hosts for the parasitic mite *Sarcoptes scabiei*, and sarcoptic mange is reported in several felid species in the scientific literature. However, the historic classification of *Sarcoptes* mites into host-specific varieties does not include *S. scabiei* var.* felis*. It is unclear whether sarcoptic mange transmission in felids involves canids, other sympatric species, or exclusively felids. This study aimed to characterize the genetic structure of *S. scabiei* mites from domestic cats (*Felis catus*) and Eurasian lynx (*Lynx lynx carpathicus*), comparing them with *Sarcoptes* mites from sympatric domestic and wild carnivores. Ten *Sarcoptes* microsatellite markers were used to genotype 81 mites obtained from skin scrapings of 36 carnivores: 4 domestic cats, one dog (*Canis lupus familiaris*), 4 Eurasian lynx, 23 red foxes (*Vulpes vulpes*), and 4 grey wolves (*Canis lupus lupus*) from either Italy, Switzerland or France. Two genetic clusters of *S. scabiei* with a geographical distribution pattern were detected: mites from cats originating from Central Italy clustered with those from sympatric wolves. In contrast, all the other mites from Switzerland, France and Northern Italy clustered together. These results strengthen the previously advanced hypothesis that genetic variants of *S. scabiei* have a predominant geographic-related distribution with cryptic transmission patterns. These patterns may rely on the interactions between different hosts living in the same ecological niche rather than a simple infection among hosts belonging to the same taxon, reinforcing the idea that the *S. scabiei* historic classification into “var” might have little ongoing relevance.

## Abstract (fr)

Les félidés domestiques et sauvages sont considérés comme des hôtes appropriés pour l'acarien parasite *Sarcoptes scabiei*, et la gale sarcoptique est signalée chez plusieurs espèces de félidés dans la littérature scientifique. Cependant, la classification traditionnelle des acariens du genre *Sarcoptes* en variétés spécifiques à l'hôte n'inclut pas* S. scabiei* var. *felis*. On ne sait pas si la transmission de la gale sarcoptique chez les félidés implique des canidés, d'autres espèces sympatriques ou exclusivement des félidés. Cette étude visait à caractériser la structure génétique des acariens *S. scabiei* des chats domestiques (*Felis catus*) et du lynx eurasien (*Lynx lynx carpathicus*), en les comparant aux *Sarcoptes* des carnivores domestiques et sauvages sympatriques. Dix marqueurs microsatellites de *Sarcoptes* ont été utilisés pour génotyper 81 acariens issus de grattages cutanés de 36 carnivores : 4 chats domestiques, un chien (*Canis lupus familiaris*), 4 lynx eurasiens, 23 renards roux (*Vulpes vulpes*) et 4 loups gris (*Canis lupus lupus*) d'Italie, de Suisse ou de France. Deux groupes génétiques de *S. scabiei*, qui suivent un modèle de distribution géographique, ont été détectés. Les acariens des chats originaires du centre de l'Italie se regroupent avec ceux des loups sympatriques. En revanche, tous les autres acariens de Suisse, de France et d'Italie du Nord sont groupés ensemble. Ces résultats renforcent l'hypothèse précédemment avancée selon laquelle les variants génétiques de *S. scabiei* ont une distribution géographique prédominante avec des schémas de transmission cryptiques. Ces modèles peuvent reposer sur les interactions entre différents hôtes vivant dans la même niche écologique plutôt que sur une simple transmission parmi des hôtes appartenant au même taxon, renforçant l'idée que la classification historique de *S. scabiei* en “var” a peu de pertinence.

## Introduction

Sarcoptic mange, also referred to as scabies, is a highly contagious skin disease caused by the burrowing mite *Sarcoptes scabiei*, affecting 200 million people each year (WHO, 2020 [45) [13, 17] and more than 150 mammal species [29]. Concerning free-ranging felids, cases have been reported in the Eurasian lynx (*Lynx lynx carpathicus*) [35], Iberian lynx (*Lynx pardinus*) [27], and European wild cat (*Felis silvestris silvestris*) [26] in Europe; Himalayan lynx (*Lynx lynx isabellinus*) [11] and snow leopard (*Uncia uncia*) (although only clinically suspected) [28] in Asia; leopard (*Panthera pardus*), lion (*Panthera leo*) and cheetah (*Acinonyx jubatus*) [10] in Eastern Africa, though not in wild felids in the Americas (Table 1).

**Table 1.** Cases of sarcoptic mange previously reported in felids.


Country	Host species	Number of individuals	Suspected origin	Reference

UK	Domestic cat (*Felis catus*)	1	Fox	[12]
Indonesia	Domestic cat (*Felis catus*)	9*	NA	[15]
Sweden	Domestic cat (*Felis catus*)	25	Dog	[4, 29, 40]
	Eurasian lynx* (Lynx lynx carpathicus)*			
Taiwan	Domestic cat (*Felis catus*)	5*	NA	[14]
Australia	Domestic cat (*Felis catus*)	4*	Dog, fox, wombat	[20]
India	Domestic cat (*Felis catus*)	1	Dog	[38]
India	Domestic cat (*Felis catus*)	1	NA	[37]
Switzerland	Eurasian lynx (*Lynx lynx carpathicus*)	2*	Fox/lynx	[35]
Norway	Eurasian lynx (*Lynx lynx carpathicus*)	NA	NA	[25]
Germany	Eurasian lynx (*Lynx lynx*)	NA	Fox	[34]
Spain	Iberian lynx (*Lynx pardinus*)	1	Fox	[27]
Pakistan	Himalayan lynx (*Lynx lynx isabellinus*)	1	Fox livestock	[11]
Pakistan	Snow leopard (*Uncia uncia*)	NA	Blue sheep	[28]
Spain	European wild cat (*Felis silvestris silvestris*)	1	Fox/cat/dog/rabbit	[26]
Kenya	Cheetah (*Acinonyx jubatus*)	3	Thomson’s gazelle	[10]
Kenya	Lion (*Panthera leo*)	3	Wildebeest	[10]
South Africa	Leopard (*Panthera pardus*)	NA	NA	[29]

NA: information not available; *Multiple cases reported in the same article.

Sarcoptic mange is considered a rare disease in domestic cats (*Felis catus*), more frequently affected by “feline mange”, a similar condition caused by the related burrowing mite *Notoedres cati* [19]. However, several confirmed sarcoptic mange cases in domestic cats have appeared in the scientific literature in the last two decades [12, 14, 15, 20, 37, 38] (Table 1), including an epidemic involving 25 animals [4]. The origin of these unusual episodes has been empirically attributed to contact with affected dogs living in the same household [20, 38] or, more rarely, with foxes visiting neighbouring gardens [12, 20].

Similarly, in wild felids, the source of *S. scabiei* transmission has been associated with sympatric hosts within carnivore communities (e.g., Eurasian lynx and red foxes (*Vulpes vulpes*) in continental Europe [5]) or in a prey-to-predator context (e.g., gazelle *Eudorcas thomsonii* and cheetahs in Eastern Africa [10]). Molecular evidence of the robustness of the latter association has been obtained using molecular markers in the case of lion, cheetah and the respective favourite ruminant preys in Masai Mara, Kenya [10].

*Sarcoptes scabiei* has been traditionally classified into host-specific varieties. Still, growing molecular evidence shows that the mere taxon-oriented approach is insufficient to embrace the issue’s complexity [7]. In this regard, various molecular tools have become available to deepen our understanding of the genetic differences between *Sarcoptes* strains affecting different host species and to track transmission pathways more efficiently and objectively. Amongst these tools, microsatellite markers have been shown to be more informative than other markers in characterizing the populations’ “strains” of *S. scabiei* affecting wildlife in Oceania [43], Europe [5, 23, 31, 36, 41], Africa [10], Asia [21], and South and North America [33, 42], sometimes revealing unexpected patterns of spread.

In line with the infrequent occurrence of sarcoptic mange in domestic cats, *Sarcoptes* isolates from feline hosts have never been characterized at a molecular level. This leaves open speculation on the possible transmission pathways and reservoir hosts and possible “strain”-specific diversity regarding pathogenicity. This study aims to investigate the molecular profile of *Sarcoptes* mites obtained from domestic and wild felids from different European countries and to compare them with *Sarcoptes* mites from sympatric and allopatric wild carnivores (Felidae and Canidae families), using microsatellite markers.

## Material and methods

Skin scrapings from four domestic cats and a dog were collected in Italy, France and Switzerland during a dermatological examination due to severe itch and crusted skin lesions. In contrast, skin samples were also obtained from wildlife with skin lesions compatible with sarcoptic mange and submitted for post-mortem examination (Table 2).

**Table 2:** Origin and sample size of the animals affected by sarcoptic mange included in this study.


Sampling site	Host species	*N*	*n*

France	Domestic cat (*Felis catus*)	1	3
Central Italy	Domestic cat (*Felis catus*)	2	8
Switzerland	Domestic cat (*Felis catus*)	1	6
Switzerland	Eurasian lynx (*Lynx lynx*)	4	8
France	Eurasian lynx (*Lynx lynx*)	1	5
Switzerland	Red fox (*Vulpes vulpes*)	11	11
North Italy	Red fox (*Vulpes vulpes*)	12	28
North Italy	Domestic dog (*Canis lupus familiaris*)	1	3
France	Wolf (*Canis lupus*)	2	5
Central Italy	Wolf (*Canis lupus*)	2	4

*N*: number of sampled animals; *n*, number of mites used for microsatellite analysis.

All samples were stored at −20 °C in 70% ethanol tubes until mite isolation and later shipped to the Department of Veterinary Sciences of Turin, Italy. Morphological criteria were applied for the preliminary identification of collected mites [8]. For each skin sample, one to six mites were isolated and individually stored in 70% ethanol [3].

DNA was extracted from individual mites following the HotSHOT Plus ThermalSHOCK technique [2]. Then, a 10x multiplex PCR was performed using ten validated primers extracted from the previously published panel [44] to target *S. scabiei* mites (Sarms 33, 34, 35, 36, 37, 38, 40, 41, 44, 45) following the PCR protocol of Soglia *et al*. [39]. Capillary electrophoresis was performed with an Applied Biosystems SeqStudio^TM^. The software GeneMapper 4.0 (Applied Biosystems, Foster City, CA, USA) allowed the allele calls and microsatellite visualization. After molecular analysis, mites that did not fulfil the required criteria (eight detectable loci out of the ten analyzed) were excluded from the genetic analysis. Two population genetics analyses were applied to the 81 mite microsatellite outputs: i) Bayesian clustering and ii) principal component analysis (PCA). The first one requires Hardy-Weinberg equilibrium (HWE), while no assumptions are required for the PCA. Descriptive statistics, such as observed and expected heterozygosis (Ho and He, respectively), allelic richness (R) and HWE analysis, were carried out with software R 4.0 using the packages Adegenet 2.1.3 [16]. *P*-values for the HWE test were based on Monte Carlo permutations of alleles. The Bayesian assignment test was computed with the software STRUCTURE 2.3.4. Burn-in and run lengths of Markov chains were 10,000 and 100,000, respectively, and five independent runs for each *K* (for *K* = 1–20) were run. The ancestry model selected was the admixture type. Clusters were estimated as suggested by Evanno [6], using the DK method.

## Results

A total of 53 alleles were detected. Allele count ranged from 2 (Sarms 34) to 11 (Sarms 45). Eleven private alleles were found across the ten microsatellite loci, distributed among six loci (Sarms 33, 34, 38, 41, 44, 45). Deviation from HWE was detected only in Sarms 34 (supplementary material, Table S1). Observed heterozygosity ranged between 0.07 (Sarms 37) and 0.88 (Sarms 41) (Table 3).

**Table 3:** Descriptive statistics of the *Sarcoptes* populations arranged by Sarms locus.


Mst locus	Ho	He

Sarms 33	0.09	0.25
Sarms 34	0.01	0.01
Sarms 35	0.03	0.52
Sarms 36	0.04	0.52
Sarms 37	0.01	0.27
Sarms 38	0.09	0.54
Sarms 40	0.04	0.23
Sarms 41	0.05	0.42
Sarms 44	0.05	0.33
Sarms 45	0.06	0.47

**Figure 1:**
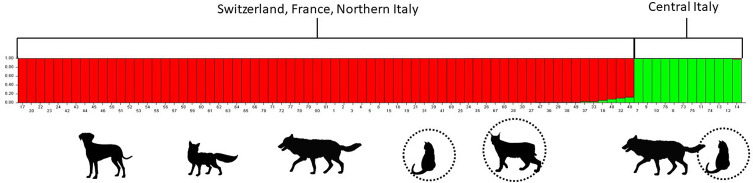
Barplot of *Sarcoptes*-derived genetic clusters generated with the software Structure 2.3.4 with maximum likelihood *K* = 2. Each mite is represented by a single bar, and the height of each coloured segment is proportional to the membership fraction in each cluster. Felids are marked with a dotted circle.

The Bayesian assignment test revealed the presence of two geographically separated clusters (Fig. 1). One cluster included mites from the cats and wolves from Central Italy (green cluster), and the other from foxes, lynx, wolves, dog and cats from France, Switzerland and Northern Italy (red cluster).

**Figure 2:**
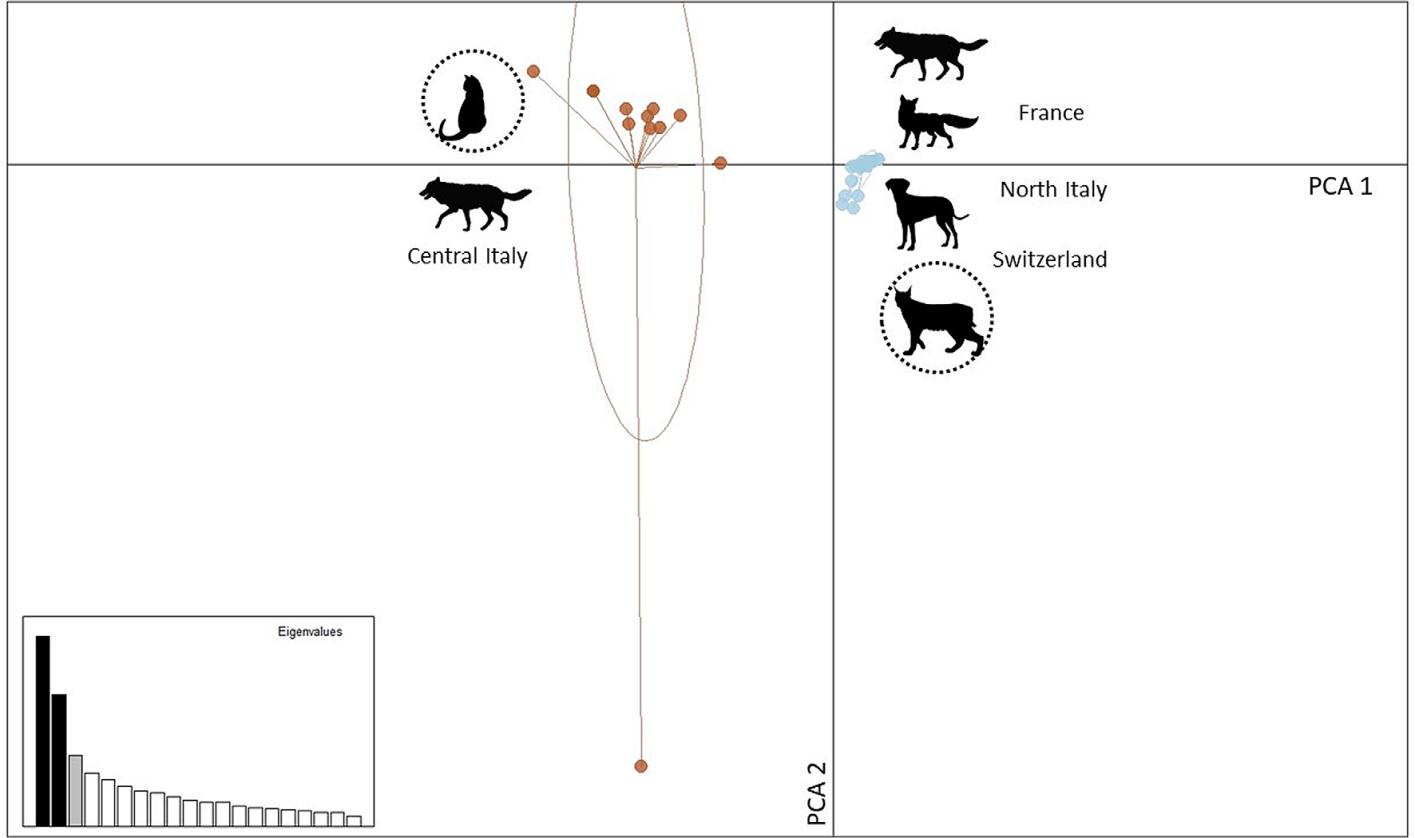
Principal components analysis (PCA) of microsatellite loci representing cat-, dog-, lynx-, wolf- and fox-derived mite populations in France, Switzerland, North Italy and Central Italy. Each population is labelled with the host species and the geographical origin. The eigenvalues of the two axes are displayed in the barplot on the left. Components PCA 1 and PCA 2 explained 11.3% and 7.9% of the variance, respectively (black bars of the eigenvalues, inset).

The results of the PCA are displayed in Figure 2. The multivariate analysis revealed two main clusters, mainly scattered along the first axis: (i) mites collected on cats and wolves from Central Italy; and (ii) mites collected on cats, dog, wolves, foxes and lynx from France, Switzerland and Northern Italy (see also Table 2).

## Discussion

The main finding of this study is the identification, using microsatellite markers, of two genetic clusters of European *S. scabiei* with a geographical distribution pattern. Cat-derived mites from Central Italy clustered with those obtained from sympatric wolves. By contrast, all the other mites from Switzerland, France and Northern Italy clustered together.

These results align with previous evidence, though referring to different animal models, that genetic lineages of *S. scabiei* have a geographic-related distribution adding to the expected host-related distribution [23]. Interspecific transmission within broad taxa (e.g., carnivores) might thus rely on direct or indirect interactions between different hosts sharing the same ecological niche [21, 23, 31].

This finding differs from the classical idea that *S. scabiei* exclusively embraces host-specific varieties. It instead supports the view that various mammal species sharing the same environment have opportunities for direct or indirect contact (through predation, scavenging, territorial fights, denning, mating, etc.), which may allow the sharing of pathogens [7, 32].

While numerous species-related varieties of *S. scabiei* have been traditionally associated with various hosts, a felid-specific variety (e.g., a putative *S. scabiei* var. “*felis*”) has never been described. The explanation is possibly the infrequent occurrence of sarcoptic mange in domestic cats, in which notoedric mange, caused by *Notoedres cati,* is predominant. Nonetheless, in the last few decades, an increasing number of sarcoptic mange cases in both domestic and wild felids have been reported [10, 12, 15, 20, 35]. These cases referred to different epidemiological scenarios in which infected dogs, foxes or wild felids’ natural prey were pinpointed as the likely source of infection (Table 1).

Recently, nuclear molecular markers such as microsatellites have been proven valuable for investigating population genetic differences and putative transmission pathways within *S. scabiei* [21, 23, 31, 33]. To the authors’ knowledge, this is the first study to apply such tools to domestic/wild felid-derived mites, indicating, on a molecular basis, what is historically reported, namely that a var. *felis* of *S. scabiei* may not exist. This conclusion needs confirmation by further molecular analyses based, in parallel, on microsatellite markers and innovative genomic techniques [9, 18, 22, 46]. A larger dataset of mites from diversified geographical areas and carnivore communities is similarly recommended.

Interestingly, Bornstein *et al*., 2004 performed a genetic characterization (although not specifying the molecular markers employed) of *Sarcoptes* mites from six out of 25 infected cats involved in a single outbreak in Sweden. These authors noted that “the mites had DNA sequences identical to *S. scabiei* from naturally infected dogs and Swedish wildlife”. These results agree with our findings (Figs. 1 and 2) and confirm that *S. scabiei* taxonomy cannot be simplified in clear-cut host-specific varieties or subspecies, as already outlined [7, 41]. Empirical information in previous studies (Table 1) suggests that dogs and foxes play a key role in the transmission of *S. scabiei* to domestic and wild felids in various scenarios, from urban to remote natural areas.

Unlike what was observed in two large wild felids in Eastern Africa (Table 1), no prey-to-predator pattern of *S. scabiei* transmission was identified in the present study. For example, the lynx mites came from the Western Alps (Table 2), where sarcoptic mange has not been reported in the main lynx prey, the roe deer (*Capreolus capreolus*), nor in the Northern chamois (*Rupicapra rupicapra*). In these areas, the disease has been detected only in smaller and rarely preyed animals, such as the red fox [25, 30] and mustelids [1].

Despite the limited sample size and the use of a single (although particularly informative) class of genetic markers, this study may be considered baseline data in the unexplored field of felid *Sarcoptes* epidemiology in felid hosts. Our results, far from suggesting that domestic cats and wolves from Central Italy infected each other by direct contact (which can be considered a rare, though possible event), show that the same *S. scabiei* strain circulates in different carnivore hosts living in the same geographical area. It seems reasonable to assume that infected dogs or red foxes represent the missing link between wolves and cats in Central Italy (see Figs. 1 and 2). Interestingly, the owners of these cats reported that they had free outdoor access and that foxes were often seen roaming near the house.

While sarcoptic mange may not always represent a threat to domestic animals, numerous cases resulted in zoonotic transmission to the owners or members of the family in contact with the infected cat [24]. Moreover, infection by *Sarcoptes* mites circulating in European carnivore communities can put additional pressure on species or populations of conservation concern, such as the Iberian lynx and the European wild cat (not included in this study). In these wild felids and other European wild felids not included in this study, the recognition of canids (and most likely the red fox) as the expected source of infection may have practical consequences in the planning of delicate conservation and management interventions, such as restocking and reintroductions.

In conclusion, our results suggest that domestic and wild felids in Italy and neighbouring countries are affected by the same *Sarcoptes* strains as those involving sympatric canids. A specific geographical pattern may reveal the transmission pathways of *Sarcoptes* mites in the investigated carnivore hosts rather than a “host species-related only” pattern. Thus, the continued use of the term “var” in the past scientific literature referring to *Sarcoptes* host-specific subspecies or “strains” may be outdated or even misleading, appearing of little relevance. Accordingly, we believe that the contemporary understanding of the broad and sometimes unexpected host associations of *S. scabiei* should cautiously lead to the dismission of the historic nomenclature based on questionable mite morphological differences and the exclusive view of strict host association [46].

## Supplementary material

The supplementary material of this article is available at https://www.parasite-journal.org/10.1051/parasite/2023012/olm. *Table S1*: Results of the Hardy–Weinberg equilibrium test at locus (rows) and populations (columns) showing P values from the Monte Carlo test. P values less than 0.05 (*) and 0.01 (**) are considered significant.
