# Supplementary material for: Sarcoptic mange in Felidae: does Sarcoptes scabiei var. felis exist? A first molecular study
Source: Parasite. 2023 Mar 31;30:11. doi: 10.1051/parasite/2023012 (PMC10069400; doi:10.1051/parasite/2023012)
Supplement: Table S1: — Results of the Hardy–Weinberg equilibrium test at locus (rows) and populations (columns) showing P values from the Monte Carlo test. P values less than 0.05 (*) and 0.01 (**) are considered significant. [file parasite-30-11-s1.pdf]

Supplementary material.

Results of the Hardy–Weinberg equilibrium test at locus (rows) and populations (columns) showing *P* values from the Monte Carlo test. *P* values less than 0.05 (*) and 0.01 (**) are considered significant.

	Switzerland	Central Italy	France	North Italy
Sarms 33	1	**	1	1
Sarms 34	1	1	1	1
Sarms 35	**	*	1	**
Sarms 36	0.083	**	0.052	**
Sarms 37	1	**	1	1
Sarms 38	**	0.056	**	1
Sarms 40	1	**	1	1
Sarms 41	1	**	**	**
Sarms 44	1	**	1	**
Sarms 45	**	**	**	*
